# Implementing a Falls Conversation Package in General Practice: A Service Evaluation

**DOI:** 10.3390/jcm15155849

**Published:** 2026-07-27

**Authors:** Savita Ramkumar, Isaa Khan, Gabrielle Oh, See Chai Carol Chan, Sreevishnu Subrahmanian, Adam Taylor, Bethan Morgan, Aron Abraham, Austen El-Osta, Azeem Majeed, Waseem Jerjes

**Affiliations:** Faculty of Medicine, Imperial College London, London W12 0BZ, UK; savita.ramkumar22@imperial.ac.uk (S.R.); isaa.khan22@imperial.ac.uk (I.K.); gabrielle.oh2@nhs.net (G.O.); carol.chan6@nhs.net (S.C.C.C.); s.subrahmanian@nhs.net (S.S.); adam.taylor39@nhs.net (A.T.); bethan.morgan23@imperial.ac.uk (B.M.); aron.abraham23@imperial.ac.uk (A.A.); a.el-osta@imperial.ac.uk (A.E.-O.); a.majeed@imperial.ac.uk (A.M.)

**Keywords:** falls prevention, general practice, service evaluation, quality improvement, older adults, patient education, patient-reported action

## Abstract

**Background**/**Objectives:** Falls-prevention advice in primary care does not always lead to a practical next step. We evaluated a brief, non-clinical Falls Conversation package at one urban NHS general practice. **Methods:** During a 12-week quality improvement/service evaluation, patients aged 65 years or older were offered a baseline reflection survey, written information, a home-safety checklist and, in later Plan-Do-Study-Act (PDSA) cycles, a one-action prompt. The package did not include clinical assessment, treatment, referral or medication review. Matched pre-post outcomes were analysed, with actions and balancing outcomes recorded at four to six weeks. **Results:** Of 510 eligible patients, 442 were approached, 334 engaged, 295 provided matched pre-post data and 256 completed follow-up. The mean project-specific knowledge score was 1.83 (SD 1.02) before and 2.60 (SD 0.95) immediately after the package, a paired difference of 0.77 out of 4 (95% CI 0.65 to 0.88; Cohen’s d_z = 0.79; *p* < 0.001). Worry changed by −0.07 out of 5 (95% CI −0.13 to −0.01; d_z = −0.13), and activity avoidance by −3.4 percentage points (95% CI −7.5 to 0.7; *p* = 0.144). At follow-up, 157/256 participants reported at least one action (61.3%, 95% CI 55.2% to 67.1%); actions were heterogeneous and unverified. Action selection and completion rose descriptively across non-randomised PDSA cohorts. **Conclusions:** The package appeared feasible to embed in one practice and was associated with higher immediate knowledge scores and patient-reported action. It did not demonstrate reduced falls or clinically meaningful change in fear-related outcomes. Controlled studies using validated measures, objective outcomes and longer follow-up are required.

## 1. Introduction

Falls threaten safety, confidence and independence in later life. International guidance treats prevention as a multidomain task that may involve strength and balance, medicines, vision, home hazards, footwear, continence, cardiovascular symptoms, fear of falling and access to specialist or community support [1,2,3,4,5]. Exercise, environmental modification and appropriately targeted multifactorial approaches can reduce falls, but their value depends on whether people can understand the advice and act on it in everyday life [2,3,6,7,8,9,10].

General practice offers repeated contact with older adults through long-term condition reviews, medicines discussions and consultations about pain, frailty, urinary symptoms, dizziness or social concerns. These contacts create opportunities to raise falls prevention before a serious injury occurs. In practice, however, time pressure, uncertainty about ownership and variable access to physiotherapy, optometry, podiatry, pharmacy and community exercise services can leave falls advice brief, generic or disconnected from a next step [11,12,13,14,15,16]. A low-burden primary-care workflow therefore needs to be understandable, repeatable and realistic within routine appointments [17,18].

The implementation gap is not solved by education alone. Knowledge may improve after an educational intervention, while sustained behaviour change remains uncertain unless the advice feels relevant and is linked to confidence, self-management and follow-up [4,5,10]. The wording also matters: prevention should support safe activity and independence rather than increase fear or withdrawal [17,18,19]. Medicines provide a further boundary. Patients may benefit from knowing that some medicines can affect balance, but review or deprescribing remains a clinician-led process outside a patient information package [20,21,22,23,24,25,26,27,28,29,30].

The Falls Conversation project was developed to test a simple awareness-to-action workflow rather than a clinical falls pathway. It combined a short reflection survey, written falls-prevention information, a home-safety checklist and a prompt to choose one practical action. This evaluation aimed to describe reach and acceptability and to examine short-term changes in a project-specific knowledge score, intention, patient-reported action and balancing outcomes among adults aged 65 years or older. It did not assess diagnostic accuracy, referral effectiveness, medication change or falls reduction.

## 2. Materials and Methods

### 2.1. Design and Setting

This was a pragmatic, 12-week local quality improvement/service evaluation conducted in a large urban NHS general practice in London with academic affiliations. The practice served approximately 19,000 registered patients, around 30% of whom were aged 65 years or older, and a socioeconomically and ethnically diverse population. The multidisciplinary team included general practitioners, practice nurses, healthcare assistants, pharmacists and administrative staff. During the implementation window, 510 attending patients aged 65 years or older met the operational eligibility criteria; this was an attendance-based implementation denominator rather than the practice’s total registered older population.

The package was delivered during routine contacts by general practitioners, practice nurses and healthcare assistants. Among engaged participants, 40.1% were in IMD quintiles 1–2. Participant-level health literacy, language need, interpreter use and the amount of staff assistance were not recorded. The practice had routine access to interpreting support for patients whose first language was not English.

### 2.2. Participants and Recruitment

Eligible patients were registered with the practice, aged 65 years or older and attended during the implementation period. They were approached opportunistically when participation would not interfere with clinical priorities. Patients were not approached when they were acutely unwell or distressed, unable to engage at that time, or when the consultation context made a falls-prevention discussion inappropriate. 

Engagement was defined as completion of the baseline reflection survey and receipt of the Falls Conversation pack. Patients could complete the survey independently or with reasonable support from practice staff. No formal sample-size calculation was undertaken because the project evaluated implementation over a prespecified 12-week period; the number of eligible attenders during that period determined the available sample.

### 2.3. Falls Conversation Package, Delivery and Project Boundary

The package comprised four elements: a brief baseline reflection survey, written falls-awareness information, a home-safety checklist and a one-action prompt. The survey covered previous falls and near-falls, strength and balance activity, night-time toileting, stairs and other home features, vision, dizziness on standing, chair-rise difficulty, foot problems, available support, worry and activity avoidance. The standard prompt was: “Which one change feels most useful for you this week?” Table 1 summarises the components and the boundary of the project. The intervention materials, staff delivery guide and project boundaries are provided in Supplementary Table S1.

The package was educational and reflective. It did not introduce physical assessment, clinical risk stratification, investigation, treatment, referral, medication review or a new follow-up pathway. Staff received brief familiarisation with the materials, their delivery sequence, the standard prompt and the distinction between education and usual clinical care. There was no formal competency assessment or fidelity audit.

No project-specific automated trigger or escalation algorithm was built into the survey. Staff were nevertheless advised to remind patients that recurrent falls, significant dizziness, concerns about medicines affecting balance or other symptoms requiring clinical attention should be discussed with their usual general practitioner or another appropriate member of the primary care team through existing NHS care pathways. Any clinical response remained part of usual care, was outside the evaluation protocol and was not captured in the project dataset.

### 2.4. Plan-Do-Study-Act Cycles

Implementation proceeded through three sequential PDSA cycles. Cycle 1 introduced the written falls-awareness information and home-safety checklist. Cycle 2 added the one-action prompt to make the transition from awareness to action explicit. Cycle 3 shortened some wording, clarified signposting and used more confidence-focused language. The cycles were not randomised, no concurrent control group was used, and the patients and staff exposure differed over time. The documented PDSA-cycle modifications are summarised in Supplementary Table S2.

### 2.5. Measures and Instrument Development

The questionnaires were developed pragmatically for this local project from the domains covered by falls-prevention guidance and the content of the package. No formal expert review, cognitive testing, pilot validation or reliability assessment was undertaken, and no minimal important difference was established. The primary outcome was a four-item project-specific knowledge composite among matched respondents. One point was assigned for each of the following: naming at least three falls-prevention actions, knowing how to check the home for trip hazards, understanding that medicines may affect balance, and knowing whom to ask for falls advice. The total score ranged from 0 to 4.

The retained analytic file recorded each knowledge item as yes/correct or no/incorrect. The free-response item, “Can you name three ways to reduce your chance of falling?”, was scored as correct when at least three distinct actions from the predefined categories were recorded: reducing home hazards or improving lighting; undertaking strength or balance activity; arranging vision assessment; using suitable footwear or seeking foot care; raising recurrent falls, near-falls, dizziness or nocturia through usual care; discussing medicines or balance concerns with a clinician or pharmacist; and identifying support, alarm or community options. Coding was undertaken by one member of the project team (WJ) using these categories. No second independent coding or inter-rater agreement assessment was performed, and the original free-text responses were not retained in the analytic dataset.

Worry about falling was recorded on a five-point scale, with higher scores indicating greater worry; “high worry” at baseline represented scores of 4 or 5. Activity avoidance was analysed as sometimes/often versus never/rarely. Immediate post-package items recorded confidence, perceived control, intention, clarity, usefulness, relevance, tailoring and increased worry as binary responses. These measures were intended for implementation learning rather than diagnosis or clinical outcome assessment.

### 2.6. Follow-Up and Outcome Definitions

A four-to-six-week interval was chosen pragmatically to give patients time to attempt a small action while remaining within the implementation period and limiting recall loss. Follow-up recorded an overall yes/no response to whether at least one falls-prevention action had been completed and then asked about specific actions. The overall measure was deliberately broad; the component actions were heterogeneous, non-mutually exclusive and varied in the degree to which they depended on self-management, routine clinical contact or access to another service. For reporting, they were grouped as home/environmental actions, activity/community actions, healthcare-contact actions, medication-related discussion and support/alarm actions.

All actions were patient-reported and were not checked against clinical records, referrals, medication reviews, home changes or exercise attendance. Falls, near-falls, increased worry and reduced activity during follow-up were recorded as balancing or safety-monitoring outcomes only. The project was not powered to evaluate falls, fractures, emergency attendance or admission.

### 2.7. Statistical Analysis

Categorical variables were summarised as counts and percentages and continuous outcomes as means and standard deviations (SD). Analyses used complete cases for each outcome; no values were imputed. Matched continuous outcomes were compared using two-sided paired *t*-tests. Because the paired differences were bounded or discrete and the matched sample was large, no formal normality test was used; the distributions were inspected for extreme outliers, and Wilcoxon signed-rank tests were undertaken as sensitivity analyses. Mean paired effects were expressed as Cohen’s d_z, calculated as the mean paired difference divided by the SD of the paired differences.

Matched binary outcomes were compared with continuity-corrected McNemar tests. Ninety-five per cent confidence intervals (CI) for paired absolute percentage-point changes were estimated by non-parametric bootstrap resampling of patient pairs (50,000 resamples). Wilson 95% CIs were used for single proportions. PDSA-cycle proportions were presented descriptively with Wilson CIs and were not compared with an inferential test because the sequential cohorts were non-randomised and not directly comparable. No adjustment for multiple comparisons was made because the analyses were exploratory; effect estimates and CIs were therefore prioritised over isolated *p*-values.

### 2.8. Governance, Consent and Data Handling

The project was managed locally as a quality improvement/service evaluation because it assessed a routine service-development package and did not allocate treatment or alter clinical care. The UK Health Research Authority “Do I need NHS REC review?” decision tool indicated that NHS Research Ethics Committee review was not required for sites in England; no IRAS Project ID was entered. Patients were informed that participation was voluntary and that their responses would be used to evaluate and improve the package. Consent was indicated by voluntary completion of the questionnaire. The analytic dataset used project identifiers and contained no direct patient identifiers. Follow-up participation was voluntary.

No separate written information sheet was used; staff provided a brief written and verbal explanation of the evaluation before questionnaire completion. Permission for one follow-up contact at four to six weeks was recorded on the project form, with contact details held separately from the analytic dataset.

## 3. Results

### 3.1. Participant Flow, Engagement and Attrition

**Figure 1:**
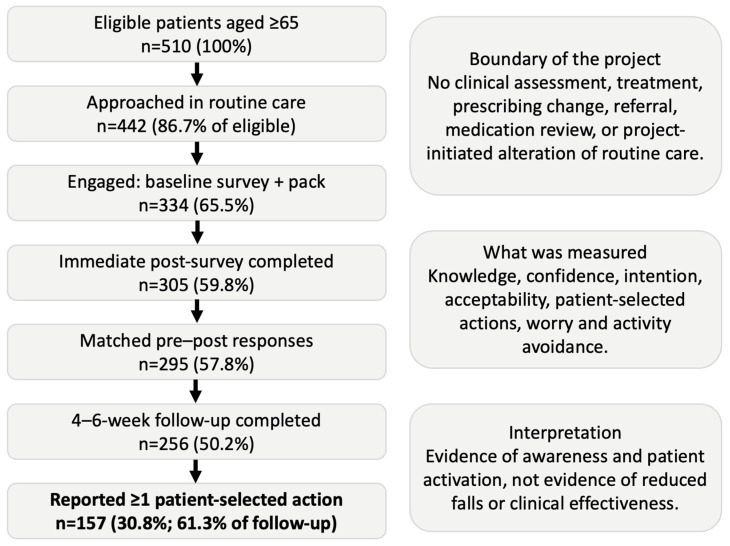
Participant flow through the Falls Conversation evaluation. Structured reasons for non-approach and non-engagement were not recorded.

During the 12-week period, 510 patients aged 65 years or older met the operational eligibility criteria. Sixty-eight were not approached; individual reasons were not recorded. Of the 442 patients approached, 334 completed the baseline survey and received the pack, giving an engagement proportion of 65.5% of all eligible patients and 75.6% of those approached. Reasons for non-engagement among the remaining 108 approached patients were not recorded. Immediate post-package data were available for 305 participants, including 295 with matched baseline and post-package responses. Follow-up was completed by 256 participants, and 157 reported at least one action. Figure 1 shows the participant flow and denominators.

The engaged and non-engaged groups had similar distributions of sex, IMD group and self-rated health, although patients aged 85 years or older accounted for 11.7% of the engaged group and 17.0% of the non-engaged group (Supplementary Table S3). Among the 334 engaged participants, 256 completed follow-up and 78 did not. The largest baseline percentage-point differences were for low or no strength-balance activity (55.9% among completers versus 43.6% among non-completers), high worry (35.5% versus 44.9%), female sex (60.2% versus 51.3%) and IMD quintiles 1–2 (41.8% versus 34.6%). The full comparison is provided in Supplementary Table S4; no missing-data weighting or modelling was applied.

### 3.2. Baseline Profile

**Figure 2:**
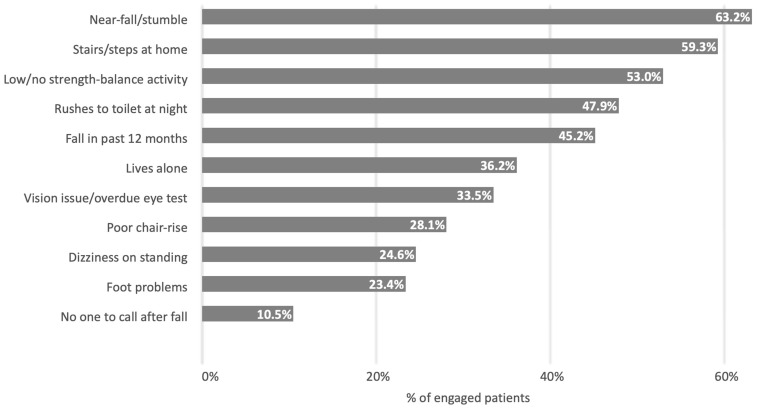
Baseline self-reported falls-related risk profile among engaged patients.

The 334 engaged participants included 194 women (58.1%), 121 people living alone (36.2%), 150 who rated their health as fair or poor (44.9%) and 134 in IMD quintiles 1–2 (40.1%). Previous falls and near-falls were common: 151 participants (45.2%) reported a fall in the preceding 12 months and 211 (63.2%) reported a near-fall or stumble. Low or no strength-balance activity was reported by 177 (53.0%), rushing to the toilet at night by 160 (47.9%) and high worry about falling by 126 (37.7%). Table 2 gives the full baseline profile, and Figure 2 displays the principal self-reported falls-related issues.

### 3.3. Matched Pre–Post Outcomes 

Among 295 matched respondents, the mean four-item knowledge score was 1.83 (SD 1.02) at baseline and 2.60 (SD 0.95) immediately after the package. The paired difference was 0.77 points (95% CI 0.65 to 0.88; Cohen’s d_z = 0.79; *p* < 0.001). Item-level absolute changes ranged from 7.5 percentage points for knowing whom to ask for advice to 26.1 percentage points for naming at least three falls-prevention actions. Wilcoxon sensitivity analyses led to the same conclusions (Supplementary Table S5).

**Figure 3:**
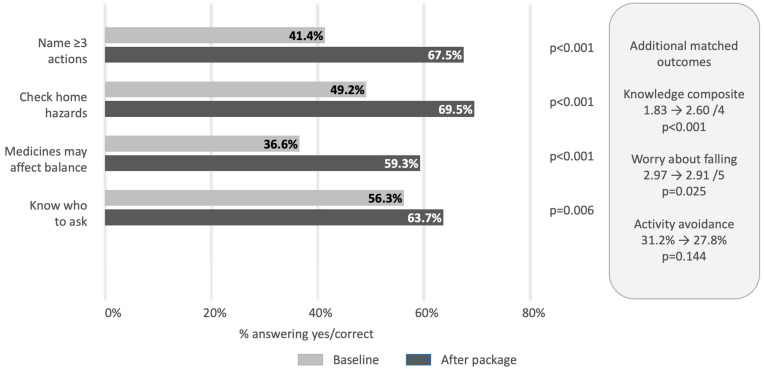
Matched pre–post proportions for four project-specific knowledge items. Paired changes and 95% confidence intervals are reported in Table 3.

Mean worry changed from 2.97 (SD 1.24) to 2.91 (SD 1.22), a paired difference of −0.07 points on the five-point scale (95% CI −0.13 to −0.01; d_z = −0.13; *p* = 0.025). Activity avoidance was reported by 92/295 participants (31.2%) at baseline and 82/295 (27.8%) after the package, a change of −3.4 percentage points (95% CI −7.5 to 0.7; *p* = 0.144). Table 3 reports the paired estimates and Figure 3 shows the four knowledge items.

### 3.4. Immediate Intention and Acceptability

Among 305 immediate post-package respondents, 252 (82.6%, 95% CI 78.0% to 86.5%) described the information as clear, 227 (74.4%, 95% CI 69.2% to 79.0%) found the checklist useful and 180 (59.0%, 95% CI 53.4% to 64.4%) felt that the information was tailored to their situation. A home change was planned by 196 participants (64.3%), strength or balance exercise by 158 (51.8%), and raising medicines or balance concerns during routine care by 117 (38.4%). Twenty-nine participants (9.5%, 95% CI 6.7% to 13.3%) reported feeling more worried immediately after reading the information. Table 4 reports all post-package items.

### 3.5. Four-to-Six-Week Follow-Up Actions and Balancing Outcomes

At four to six weeks, 157/256 respondents reported at least one falls-prevention action (61.3%, 95% CI 55.2% to 67.1%). The most frequently reported components were removing clutter, securing rugs or changing layout (91/256, 35.5%), starting or increasing strength/balance activity (73/256, 28.5%) and improving lighting or the night-time toilet route (65/256, 25.4%). Healthcare-contact actions included mentioning dizziness, a near-fall or nocturia during routine care (48/256, 18.8%) and booking or completing an eye test or glasses review (46/256, 18.0%). Thirty-eight participants (14.8%) reported that they had raised medicines or balance concerns during routine care. Two participants answered yes to the overall action item but did not select any listed component, indicating another or unspecified action. Table 5 groups the components by action type; participants could report more than one action.

During the follow-up period, 18/256 participants (7.0%) reported a fall and 42/256 (16.4%) a near-fall. Twenty-two (8.6%) felt more worried because of the information and 15 (5.9%) reported doing less activity because of falls concern. These were descriptive balancing outcomes and were not evidence of a change in fall risk.

### 3.6. Descriptive PDSA-Cycle Trends

**Figure 4:**
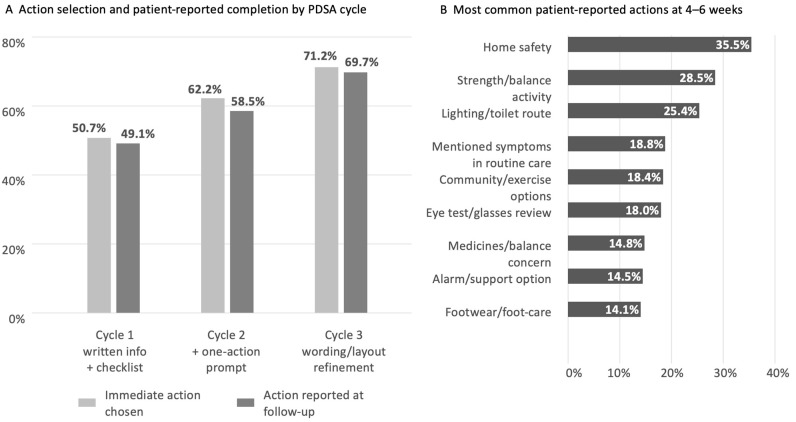
Descriptive proportions for immediate action selection and patient-reported action at follow-up across sequential PDSA cycles, with the follow-up action profile. The cycle cohorts were non-randomised and not directly comparable.

The proportion selecting an immediate action was 35/69 in Cycle 1 (50.7%, 95% CI 39.2% to 62.2%), 69/111 in Cycle 2 (62.2%, 95% CI 52.9% to 70.6%) and 89/125 in Cycle 3 (71.2%, 95% CI 62.7% to 78.4%). Patient-reported action at follow-up was 26/53 (49.1%, 95% CI 36.1% to 62.1%), 55/94 (58.5%, 95% CI 48.4% to 67.9%) and 76/109 (69.7%, 95% CI 60.5% to 77.6%), respectively. These were descriptive sequential trends; no between-cycle significance test was performed (Supplementary Table S6). Figure 4 shows the cycle proportions and the follow-up action profile.

## 4. Discussion

### 4.1. Principal Findings

In this single-practice service evaluation, a brief Falls Conversation package was delivered to 334 of 510 eligible patients and was associated with higher immediate scores on a four-item, project-specific knowledge measure. At four to six weeks, 61.3% of follow-up respondents reported at least one action. The evaluation did not show a clinically meaningful change in worry, a clear change in activity avoidance or any reduction in falls. Its contribution is therefore implementation and educational learning rather than evidence of clinical effectiveness.

The mean knowledge difference of 0.77 points represented 19.2% of the four-point scale range, and the standardised paired effect was d_z = 0.79. That magnitude appears substantial statistically, but it should not be treated as an established clinically important effect. The scale was developed for this project, had no validated minimal important difference, and was repeated immediately after participants had seen closely related information. The observed change may reflect learning, but it may also include recall, baseline-question priming, additional attention from staff and social desirability. A delayed assessment or controlled comparison would be needed to separate these explanations.

Fear-related outcomes provide a useful counterweight to the knowledge findings. The mean worry change was only −0.07 on a five-point scale (d_z = −0.13), and the confidence interval was close to zero. Activity avoidance did not show a clear paired change. The most defensible interpretation is that the package was not associated with a large short-term worsening of these measures among respondents; the data do not support a meaningful improvement in fear of falling.

### 4.2. Implementation Learning and Meaning of Reported Action

The one-action prompt addressed a practical problem in primary care: advice is easier to act on when it ends with a specific, manageable step. The rising cycle-level proportions are consistent with that implementation logic, but they cannot establish that the prompt or later wording changes caused the trend. The cycles involved different patients, staff experience accumulated over time, and recruitment and follow-up could have changed. The trends should therefore guide further testing rather than be read as comparative effectiveness results.

The overall action outcome also requires careful interpretation. Home changes, exercise, eye care, symptom discussion, medicines discussion and support planning differ in effort, access requirements and clinical significance. Grouping the components makes clear that “at least one action” is a broad marker of patient-reported activation, not a uniform clinical endpoint. None of the actions was independently verified, and two respondents reported another or unspecified action. The follow-up results consequently describe what participants said they had done, not confirmed behaviour or service use.

The medicines items illustrate the boundary particularly well. Higher awareness that medicines may affect balance may prompt a useful conversation, but it is not evidence that medicines were reviewed, changed or deprescribed. At follow-up, 38 participants reported raising medicines or balance concerns during routine care. Any subsequent decision belongs within clinician-led review, shared decision-making and attention to competing indications [21,22,24,25]. A pharmacist-supported component would be a separate clinical intervention and should be evaluated as such.

Most immediate acceptability items had high yes-response proportions, particularly clarity and willingness to mention a near-fall. Binary response options may have produced ceiling effects and limited the ability to distinguish between adequate and excellent acceptability. The four-to-six-week interval was long enough for an initial action but too short to assess persistence, repeated action or effects on falls. Future work should use more discriminating response scales and repeated follow-up.

### 4.3. Strengths and Limitations 

Strengths include transparent reporting of participant flow, a clear boundary between education and clinical care, paired analyses, confidence intervals, a high follow-up proportion among immediate respondents and explicit balancing outcomes. The package used existing staff and a short routine-care workflow, which makes the implementation question relevant to general practice. Reporting the PDSA changes and their limits also provides information that a later study can test more rigorously.

The main limitation is the uncontrolled pre-post design. Testing effects, priming, Hawthorne effects and social desirability cannot be separated from the package itself. The measures were pragmatic and unvalidated; the retained dataset did not allow retrospective assessment of free-response coding reliability. Immediate post-package responses are particularly vulnerable to recall and courtesy bias. No formal fidelity audit was undertaken, and the project did not record the duration of delivery, the amount of staff help or variation between staff members.

Selection and attrition also matter. Reasons for non-approach and non-engagement were not captured, patients aged 85 years or older were less represented among those who engaged, and follow-up completers differed from non-completers on several baseline characteristics. The evaluation did not record health literacy, cognitive impairment, sensory impairment, language need or the type of assistance required. Older adults facing these barriers, severe frailty, social isolation or limited access to community resources may have had different experiences. The single urban practice had academic affiliations, and the absence of participant-level information on language need, health literacy, interpreter use and the support required to complete the package limits transferability to rural, smaller or less-resourced settings.

All follow-up actions were self-reported and were not verified through records, referrals, medication reviews, home visits or exercise attendance. The project had no standardised safety-netting algorithm and did not capture usual-care responses to clinically concerning disclosures. Follow-up was short, falls and near-falls were monitored only descriptively, and the study was not powered for fractures, emergency attendance, admission or other clinical outcomes. The findings must not be interpreted as evidence that the package reduced falls or injuries.

### 4.4. Implications for Further Work

A next-stage evaluation should preserve the low-burden workflow while strengthening measurement and safety. Priorities include co-designed and accessible materials, translated or interpreter-supported delivery, an explicit safety-netting script, a brief fidelity measure and validated outcomes. A controlled design, delayed knowledge assessment and longer follow-up would help distinguish immediate recall from sustained learning. Objective verification through routine records could assess whether reported discussions, eye care, medication review or referrals occurred.

Testing across practices with different deprivation profiles, workforce capacity, rurality and language needs is necessary before wider implementation claims are made. If the aim extends to medication change, supervised exercise, environmental assessment or referral completion, those elements should be specified and evaluated as clinical interventions. Falls, fractures and healthcare use require adequately powered controlled studies rather than inference from a short service evaluation.

## 5. Conclusions

In one urban general practice, the Falls Conversation package appeared practical to embed in routine care and was associated with higher immediate project-specific knowledge scores and later patient-reported actions. The evaluation did not demonstrate reduced falls, a clinically meaningful reduction in worry or that the PDSA refinements caused the cycle-level trends. Replication in diverse practices using validated measures, objective outcomes, longer follow-up and controlled designs is needed before wider implementation claims can be made.

## Figures and Tables

**Table 1:** Components and boundaries of the Falls Conversation package.

Component	What Patients Received or Completed	Purpose	Boundary of the Project
Baseline reflection survey	Questions on falls, near-falls, activity, home features, vision, dizziness, night-time toileting, feet, worry and support	Prompt reflection on potentially modifiable issues	Not a clinical assessment or risk score
Written falls-awareness information	Brief information about common falls risks and prevention approaches	Support awareness and understanding	Not individualised medical advice
Home-safety checklist	Prompts about clutter, rugs, lighting, stairs, footwear and night-time routes	Support patient-led review of the home	No home visit or professional assessment
One-action prompt	“Which one change feels most useful for you this week?”	Encourage one realistic patient-selected step	No clinician-directed treatment
Immediate post-package survey	Project-specific knowledge, confidence, intention and acceptability items	Assess immediate educational and implementation outcomes	Not a validated patient-reported outcome measure
Four-to-six-week follow-up	Self-report of actions, falls, near-falls, worry and activity avoidance	Describe patient-reported action and balancing outcomes	No verification through clinical records or referrals

**Table 2:** Baseline characteristics and self-reported falls-related profile among engaged participants (n = 334).

Domain	Characteristic	n/N (%)
Age	65–69 years	86/334 (25.7%)
Age	70–74 years	82/334 (24.6%)
Age	75–79 years	73/334 (21.9%)
Age	80–84 years	54/334 (16.2%)
Age	85 years or older	39/334 (11.7%)
Sex	Female	194/334 (58.1%)
Sex	Male	140/334 (41.9%)
Living context	Lives alone	121/334 (36.2%)
Living context	Residential care	13/334 (3.9%)
Self-rated health	Fair/poor	150/334 (44.9%)
Equity marker	IMD quintiles 1–2	134/334 (40.1%)
Falls-related experience	Fall in previous 12 months	151/334 (45.2%)
Falls-related experience	Near-fall/stumble in previous 12 months	211/334 (63.2%)
Activity	Low/no strength-balance activity	177/334 (53.0%)
Home/toileting	Rushes to toilet at night	160/334 (47.9%)
Home/toileting	Stairs/steps at home	198/334 (59.3%)
Vision	Vision issue or overdue eye test	112/334 (33.5%)
Symptoms/function	Dizziness on standing	82/334 (24.6%)
Symptoms/function	Poor chair-rise	94/334 (28.1%)
Feet	Foot problems	78/334 (23.4%)
Support	No one to call after a fall	35/334 (10.5%)
Worry	High worry about falling	126/334 (37.7%)

**Table 3:** Matched pre–post changes in project-specific knowledge and fear-related outcomes (n = 295).

Outcome	Baseline	Immediately After	Paired Change (95% CI)	Effect/Test
Knowledge composite, 0–4, mean (SD)	1.83 (1.02)	2.60 (0.95)	+0.77 (0.65 to 0.88)	d_z = 0.79; paired *t*-test *p* < 0.001
Can name at least three falls-prevention actions	122/295 (41.4%)	199/295 (67.5%)	+26.1 pp (20.3 to 31.9)	McNemar *p* < 0.001
Knows how to check the home for trip hazards	145/295 (49.2%)	205/295 (69.5%)	+20.3 pp (14.6 to 26.1)	McNemar *p* < 0.001
Understands that medicines may affect balance	108/295 (36.6%)	175/295 (59.3%)	+22.7 pp (16.9 to 28.5)	McNemar *p* < 0.001
Knows whom to ask for falls advice	166/295 (56.3%)	188/295 (63.7%)	+7.5 pp (2.4 to 12.5)	McNemar *p* = 0.006
Worry about falling, 1–5, mean (SD)	2.97 (1.24)	2.91 (1.22)	−0.07 (−0.13 to −0.01)	d_z = −0.13; paired *t*-test *p* = 0.025
Avoids activity because of fear: sometimes/often	92/295 (31.2%)	82/295 (27.8%)	−3.4 pp (−7.5 to 0.7)	McNemar *p* = 0.144

CI for continuous paired changes used the t distribution. CI for paired percentage-point changes used 50,000-resample paired bootstrap. d_z denotes Cohen’s standardised paired effect. All analyses were exploratory.

**Table 4:** Immediate post-package intention, confidence and acceptability (n = 305).

Outcome	n/N (%)	95% CI
Feels confident they could reduce their chance of falling	191/305 (62.6%)	57.1% to 67.9%
Feels more in control of their health in relation to falls	183/305 (60.0%)	54.4% to 65.3%
Plans to make one home change this week	196/305 (64.3%)	58.7% to 69.4%
Plans to try strength or balance exercise	158/305 (51.8%)	46.2% to 57.4%
Intends to raise medicines or balance concerns during routine care	117/305 (38.4%)	33.1% to 43.9%
Would mention a near-fall or stumble during routine care	236/305 (77.4%)	72.4% to 81.7%
Falls information was clear and easy to understand	252/305 (82.6%)	78.0% to 86.5%
Home-safety checklist was useful	227/305 (74.4%)	69.2% to 79.0%
Advice felt relevant	215/305 (70.5%)	65.1% to 75.3%
Information felt tailored to their situation	180/305 (59.0%)	53.4% to 64.4%
Felt more worried immediately after reading the information	29/305 (9.5%)	6.7% to 13.3%

All items were binary project-specific responses; the confidence intervals are Wilson intervals.

**Table 5:** Four-to-six-week patient-reported actions and balancing outcomes (n = 256).

Category	Patient-Reported Outcome	n/N (%)	95% CI
Overall	Reported at least one falls-prevention action	157/256 (61.3%)	55.2% to 67.1%
Home/environment	Removed clutter, secured rugs, or changed layout	91/256 (35.5%)	29.9% to 41.6%
Home/environment	Improved lighting or the night-time toilet route	65/256 (25.4%)	20.4% to 31.1%
Activity/community	Started/increased strength-balance activity	73/256 (28.5%)	23.3% to 34.3%
Healthcare contact	Booked/completed eye test or glasses review	46/256 (18.0%)	13.8% to 23.1%
Medication discussion	Raised medicines or balance concerns during routine care	38/256 (14.8%)	11.0% to 19.7%
Healthcare contact	Mentioned dizziness, a near-fall or nocturia during routine care	48/256 (18.8%)	14.4% to 24.0%
Healthcare contact	Changed footwear, checked feet or sought foot-care advice	36/256 (14.1%)	10.3% to 18.9%
Support/alarm	Discussed a falls alarm or other support option	37/256 (14.5%)	10.7% to 19.3%
Activity/community	Looked for or asked about local exercise/community support	47/256 (18.4%)	14.1% to 23.6%
Safety monitoring	Reported a fall during follow-up	18/256 (7.0%)	4.5% to 10.8%
Safety monitoring	Reported a near-fall during follow-up	42/256 (16.4%)	12.4% to 21.4%
Balancing outcome	Felt more worried because of information	22/256 (8.6%)	5.7% to 12.7%
Balancing outcome	Reported doing less activity because of falls concern	15/256 (5.9%)	3.6% to 9.4%

Action components were non-mutually exclusive. Two respondents reported an overall action without selecting a listed component. Confidence intervals are Wilson intervals.

## Data Availability

The individual-level dataset is not publicly available because of patient confidentiality and local information-governance requirements. A de-identified dataset may be made available by the corresponding author upon reasonable request, subject to institutional approval and applicable data-protection requirements.

## Supplementary Materials

The following supporting information can be downloaded at: https://www.mdpi.com/article/10.3390/jcm15155849/s1.
