# Supplementary material for: Implementing a Falls Conversation Package in General Practice: A Service Evaluation
Source: J Clin Med. 2026 Jul 27;15(15):5849. doi: 10.3390/jcm15155849 (PMC13466697; doi:10.3390/jcm15155849)
Supplement: Supplementary file 1 [file jcm-15-05849-s001.zip › jcm-4443712-supplementary.pdf]

Supplementary File S1

Implementing a Falls Conversation Package in General Practice: A Service Evaluation

S1. Falls Conversation Intervention Materials

S1.1. Patient-facing falls-prevention information

Small steps that may reduce your chance of falling

Falls can have many causes. This information is intended to help you notice common risks and choose one practical step. It provides general information and does not replace an individual clinical assessment.

Keep moving safely

- Strength and balance activity can help you stay steady. Choose an activity that is suitable for you and seek advice through usual care if you are unsure what is safe.
- Do not stop normal activity solely because you are worried about falling; seek clinical advice if fear is limiting what you do.

Make the home easier to move around

- Remove clutter from routes used often, secure loose rugs and keep stairs and steps clear.
- Check that lighting is adequate, particularly between the bed and toilet at night.
- Keep commonly used items within easy reach and wear footwear that fits securely.

Notice symptoms and health needs

- Arrange an eye test if vision has changed or a routine test is overdue.
- Raise recurrent falls, near-falls, dizziness on standing, rushing to the toilet at night or foot problems through usual primary care.
- Some medicines can affect alertness or balance. Do not stop medicines yourself; discuss concerns with a clinician or pharmacist.

Plan support and choose one action

- Think about whom you would contact after a fall and whether an alarm or another support option may be useful.
- Choose one change that feels realistic this week.

Standard one-action prompt: “Which one change feels most useful for you this week?”

S1.2. Home-safety checklist

Area	Prompt	Response
Walking routes	Floors and routes are clear of clutter, cables and bags	Yes / Not yet / Not applicable
Rugs and mats	Loose rugs or mats are removed, secured or have non-slip backing	Yes / Not yet / Not applicable
Lighting	Hallways, stairs, entrances and the night-time toilet route are well lit	Yes / Not yet / Not applicable
Stairs and steps	Steps are clear; handrails and step edges are easy to see	Yes / Not yet / Not applicable
Bathroom	Frequently used items are within reach and surfaces are as slip-resistant as possible	Yes / Not yet / Not applicable
Bedroom/night-time route	A light is within reach and the route to the toilet is clear	Yes / Not yet / Not applicable
Footwear and feet	Footwear fits securely; painful feet or nail problems are raised through usual care	Yes / Not yet / Not applicable
Vision	Glasses are suitable and an eye test is arranged if due	Yes / Not yet / Not applicable
Support	I know whom I could contact after a fall or near-fall	Yes / Not yet / Not applicable
My one action	The first change I will try is: ______________________________	Free text

S1.3. Staff delivery and safety-netting guide

1. Confirm that the patient is registered with the practice, aged 65 years or older, and that participation will not interfere with an urgent or sensitive clinical priority.
2. Explain that the package is voluntary, brief and intended for local service improvement. It is not a falls assessment, treatment pathway or substitute for clinical care.
3. Ask the patient to complete the baseline reflection and knowledge items, with reasonable support from practice staff where required.
4. Provide the written falls-prevention information and home-safety checklist.

5. Use the standard prompt: “Which one change feels most useful for you this week?”
6. Ask the patient to complete the immediate post-package items.
7. Explain that voluntary follow-up may occur at four to six weeks through the locally agreed contact process.
8. Continue to use ordinary professional judgement. The evaluation does not prevent a patient from raising symptoms or requesting review through usual care.

Safety-netting instruction. Patients reporting recurrent falls, significant dizziness, concern that medicines were affecting balance, or another symptom requiring clinical attention were advised to discuss this with their usual general practitioner or another appropriate member of the primary care team through existing NHS care pathways. Participation did not itself trigger additional referral, investigation or treatment; subsequent clinical management remained part of routine care.

Project boundary. The package did not provide physical assessment, clinical risk stratification, investigation, treatment, referral, medication review or a project-specific clinical follow-up pathway.

Training and fidelity. Staff received brief familiarisation with the materials, delivery sequence, standard prompt and project boundary. No formal competency assessment or fidelity audit was undertaken.

S2. Project-Specific Measures and Coding Framework

The instruments were developed pragmatically for this local evaluation and were not formally validated. The wording below corresponds to the retained analytic fields; response options and coding are shown explicitly.

Table S1A. Baseline reflection and profile items

Analytic item	Analytic wording	Response/coding
Fall in previous 12 months	Have you had a fall in the last 12 months?	Yes/No
Near-fall or stumble	Have you had a near-fall or stumble in the last 12 months?	Yes/No
Strength/balance activity	Do you undertake regular activity intended to improve strength or balance?	Low/none versus some/regular
Night-time toileting	Do you sometimes rush to the toilet at night?	Yes/No
Stairs or steps	Do you have stairs or steps at home?	Yes/No
Vision	Do you have a vision problem or an overdue eye test?	Yes/No
Dizziness	Do you feel dizzy when standing up?	Yes/No
Chair-rise	Do you have difficulty rising from a chair?	Yes/No
Feet	Do you have foot problems that affect walking or footwear?	Yes/No
Living context	Do you live alone, with a partner/family, or in residential care?	Three categories
Support	Is there no one you could contact after a fall?	Yes/No
Worry about falling	How worried are you about falling?	1-5; high worry = 4 or 5
Activity avoidance	Do you avoid activity because of fear of falling?	Sometimes/often versus never/rarely
Self-rated health	How would you rate your health?	Excellent/very good; good; fair/poor

Table S1B. Knowledge items administered before and immediately after the package

Item	Operational definition	Coding
Name at least three falls-prevention actions	Free response; scored correct when at least three distinct acceptable actions were recorded	1 = correct; 0 = incorrect/no
Check the home for trip hazards	Knows how to check the home for trip hazards	1 = yes/correct; 0 = no/incorrect
Medicines and balance	Understands that medicines may affect balance	1 = yes/correct; 0 = no/incorrect
Whom to ask	Knows whom to ask for falls advice	1 = yes/correct; 0 = no/incorrect
Knowledge composite	Sum of the four binary items	0-4

Free-response coding framework. Acceptable actions were drawn from the content of the package and included: reducing home hazards or improving lighting; undertaking strength or balance activity; arranging vision assessment; using suitable footwear or seeking foot care; raising recurrent falls, near-falls, dizziness or nocturia through usual care; discussing medicines or balance concerns with a clinician or pharmacist; and identifying support, alarm or community options. At least three distinct actions were required for a correct response.

Coding process. Coding was undertaken by one member of the project team using the predefined categories. The retained analytic record did not identify the coder by initials, the original free-text responses were not retained in the analysis file, and no second independent coding or inter-rater agreement statistic was available.

Table S1C. Immediate post-package items

Retained field	Analytic wording	Response
Confident_Reduce_Falls	Feels confident they could reduce their chance of falling	Yes/No
More_In_Control	Feels more in control of their health in relation to falls	Yes/No
Plans_Home_Change	Plans to make one home change this week	Yes/No
Plans_Exercise	Plans to try strength or balance exercise	Yes/No
Plans_Medicines_Balance_Concern	Intends to raise medicines or balance concerns during routine care	Yes/No
Would_Mention_Nearfall_Routine_Care	Would mention a near-fall or stumble during routine care	Yes/No
Information_Clear	Falls information was clear and easy to understand	Yes/No
Checklist_Useful	Home-safety checklist was useful	Yes/No
Advice_Relevant	Advice felt relevant	Yes/No
Tailored_To_Situation	Information felt tailored to their situation	Yes/No

Retained field	Analytic wording	Response
More_Worried_Post	Felt more worried immediately after reading the information	Yes/No

Table S1D. Four-to-six-week follow-up items

Retained field	Analytic wording	Response/coding
Completed_Any_Action	Reported completing at least one falls-prevention action	Yes/No
Home_Safety_Action	Removed clutter, secured rugs or changed layout	Yes/No
Lighting_Toilet_Route	Improved lighting or the night-time toilet route	Yes/No
Strength_Balance_Exercise	Started or increased strength/balance activity	Yes/No
Eye_Test_Action	Booked or completed an eye test or glasses review	Yes/No
Medicines_Balance_Concern_Action	Raised medicines or balance concerns during routine care	Yes/No
Mentioned_Symptoms_Routine_Care	Mentioned dizziness, a near-fall or nocturia during routine care	Yes/No
Foot_Care_Action	Changed footwear, checked feet or sought foot-care advice	Yes/No
Alarm_Support_Action	Discussed a falls alarm or another support option	Yes/No
Community_Exercise_Options_Action	Looked for or asked about local exercise/community options	Yes/No
Fall_During_Followup	Reported a fall during follow-up	Yes/No; safety monitoring
Near_Fall_During_Followup	Reported a near-fall during follow-up	Yes/No; safety monitoring
Less_Activity_Followup	Reported doing less activity because of falls concern	Yes/No; balancing outcome
More_Worried_Followup	Felt more worried because of the information	Yes/No; balancing outcome

Two of the 157 respondents who marked the overall action item "yes" did not select any listed component. They were retained in the overall outcome and described as having completed another or unspecified action.

S3. Plan-Do-Study-Act Change Log

Table S2A. Documented intervention changes by PDSA cycle

Cycle	Weeks	Documented change	Implementation rationale	Flow denominators
Cycle 1	Weeks 1-3	Written falls-awareness information and home-safety checklist	Establish whether the written package could be delivered during routine care	117 eligible; 76 engaged; 69 immediate post; 53 follow-up
Cycle 2	Weeks 4-7	Added the one-action prompt	Make the transition from information to a patient-selected next step explicit	180 eligible; 121 engaged; 111 immediate post; 94 follow-up
Cycle 3	Weeks 8-12	Shortened wording, clarified signposting and used more confidence-focused language	Improve readability and reduce passive or fear-inducing wording	213 eligible; 137 engaged; 125 immediate post; 109 follow-up

The retained project archive did not contain version-controlled copies showing every wording and layout change made in Cycle 3; the table reports the documented change summary available in the dataset and manuscript.

Table S2B. Descriptive action proportions by PDSA cycle

Cycle	Immediate action selected	Patient-reported action at follow-up
Cycle 1	35/69 (50.7%; 95% CI 39.2% to 62.2%)	26/53 (49.1%; 95% CI 36.1% to 62.1%)
Cycle 2	69/111 (62.2%; 95% CI 52.9% to 70.6%)	55/94 (58.5%; 95% CI 48.4% to 67.9%)
Cycle 3	89/125 (71.2%; 95% CI 62.7% to 78.4%)	76/109 (69.7%; 95% CI 60.5% to 77.6%)

The cycles were sequential, involved different participants and were not randomised. No inferential between-cycle test was used.

S4. Additional Participant-Flow Analyses

Table S3. Baseline demographic comparison of engaged and non-engaged eligible patients

Characteristic	Engaged (n = 334)	Not engaged (n = 176)	Difference, percentage points
Age 65-69 years	86/334 (25.7%)	39/176 (22.2%)	+3.6
Age 70-74 years	82/334 (24.6%)	38/176 (21.6%)	+3.0
Age 75-79 years	73/334 (21.9%)	40/176 (22.7%)	-0.9
Age 80-84 years	54/334 (16.2%)	29/176 (16.5%)	-0.3
Age 85 years or older	39/334 (11.7%)	30/176 (17.0%)	-5.4
Female	194/334 (58.1%)	100/176 (56.8%)	+1.3
Lives alone	121/334 (36.2%)	70/176 (39.8%)	-3.5
Residential care	13/334 (3.9%)	1/176 (0.6%)	+3.3
Fair/poor self-rated health	150/334 (44.9%)	83/176 (47.2%)	-2.2
IMD quintiles 1-2	134/334 (40.1%)	73/176 (41.5%)	-1.4

Only demographic and contextual fields were available for patients who did not engage; falls-related baseline items were collected after engagement. Differences are descriptive and were not significance-tested.

Table S4. Baseline comparison of follow-up completers and non-completers among engaged participants

Characteristic	Follow-up completed (n = 256)	No follow-up (n = 78)	Difference, percentage points
Age 65-69 years	68/256 (26.6%)	18/78 (23.1%)	+3.5
Age 70-74 years	59/256 (23.0%)	23/78 (29.5%)	-6.4
Age 75-79 years	58/256 (22.7%)	15/78 (19.2%)	+3.4
Age 80-84 years	39/256 (15.2%)	15/78 (19.2%)	-4.0
Age 85 years or older	32/256 (12.5%)	7/78 (9.0%)	+3.5
Female	154/256 (60.2%)	40/78 (51.3%)	+8.9
Lives alone	97/256 (37.9%)	24/78 (30.8%)	+7.1
Residential care	12/256 (4.7%)	1/78 (1.3%)	+3.4
Fair/poor self-rated health	112/256 (43.8%)	38/78 (48.7%)	-5.0
IMD quintiles 1-2	107/256 (41.8%)	27/78 (34.6%)	+7.2
Fall in previous 12 months	119/256 (46.5%)	32/78 (41.0%)	+5.5
Near-fall/stumble in previous 12 months	160/256 (62.5%)	51/78 (65.4%)	-2.9
Low/no strength-balance activity	143/256 (55.9%)	34/78 (43.6%)	+12.3
Rushes to toilet at night	127/256 (49.6%)	33/78 (42.3%)	+7.3
Stairs/steps at home	150/256 (58.6%)	48/78 (61.5%)	-2.9
Vision issue/overdue eye test	88/256 (34.4%)	24/78 (30.8%)	+3.6
Dizziness on standing	61/256 (23.8%)	21/78 (26.9%)	-3.1
Poor chair-rise	68/256 (26.6%)	26/78 (33.3%)	-6.8
Foot problems	60/256 (23.4%)	18/78 (23.1%)	+0.4
No one to call after a fall	31/256 (12.1%)	4/78 (5.1%)	+7.0
High worry about falling	91/256 (35.5%)	35/78 (44.9%)	-9.3

The comparison is restricted to the 334 participants who engaged with the package. Positive differences indicate a higher proportion among follow-up completers. No weighting, imputation or significance testing was used.

S5. Paired Transition Counts and Sensitivity Analyses

Table S5. Paired outcome details

Part A. Binary transition counts

Outcome	No-No	No-Yes	Yes-No	Yes-Yes
Can name at least three falls-prevention actions	87	86	9	113
Knows how to check the home for trip hazards	75	75	15	130
Understands that medicines may affect balance	109	78	11	97
Knows whom to ask for falls advice	89	40	18	148
Activity avoidance: sometimes/often	189	14	24	68

For knowledge items, No-Yes represents improvement and Yes-No represents worsening. For activity avoidance, No-Yes represents increased avoidance and Yes-No represents reduced avoidance.

Part B. Binary paired changes and McNemar tests

Outcome	Paired change, percentage points (95% CI)	McNemar p value
Can name at least three falls-prevention actions	+26.1 (20.3 to 31.9)	<0.001
Knows how to check the home for trip hazards	+20.3 (14.6 to 26.1)	<0.001
Understands that medicines may affect balance	+22.7 (16.9 to 28.5)	<0.001
Knows whom to ask for falls advice	+7.5 (2.4 to 12.5)	0.006
Activity avoidance: sometimes/often	-3.4 (-7.5 to 0.7)	0.144

Confidence intervals for paired percentage-point changes used 50,000-resample non-parametric bootstrap resampling of patient pairs. McNemar tests used the continuity correction.

Part C. Continuous paired outcomes and non-parametric sensitivity analyses

Outcome	Mean paired difference	95% CI	Cohen d_z	Paired t-test p	Wilcoxon p
Knowledge composite, 0-4	+0.77	0.65 to 0.88	0.79	<0.001	<0.001
Worry about falling, 1-5	-0.07	-0.13 to -0.01	-0.13	0.025	0.025

The Wilcoxon signed-rank sensitivity analyses led to the same inferential conclusions as the paired t-tests. No validated minimal important difference was available for either project-specific scale.

S6. PDSA-Cycle Denominators and Confidence Intervals

Table S6. Cycle-specific denominators and Wilson confidence intervals

Cycle	Eligible	Engaged	Immediate action selected	Patient-reported action at follow-up
Cycle 1	117	76	35/69 (50.7%; 95% CI 39.2% to 62.2%)	26/53 (49.1%; 95% CI 36.1% to 62.1%)
Cycle 2	180	121	69/111 (62.2%; 95% CI 52.9% to 70.6%)	55/94 (58.5%; 95% CI 48.4% to 67.9%)
Cycle 3	213	137	89/125 (71.2%; 95% CI 62.7% to 78.4%)	76/109 (69.7%; 95% CI 60.5% to 77.6%)

These estimates are descriptive. The sequential cohorts were not directly comparable, and no between-cycle p value is reported.
